# Antennal Protein Profile in Honeybees: Caste and Task Matter More Than Age

**DOI:** 10.3389/fphys.2018.00748

**Published:** 2018-06-20

**Authors:** Immacolata Iovinella, Federico Cappa, Alessandro Cini, Iacopo Petrocelli, Rita Cervo, Stefano Turillazzi, Francesca R. Dani

**Affiliations:** ^1^Department of Biology, Università degli Studi di Firenze, Florence, Italy; ^2^Centre for Biodiversity and Environment Research, University College London, London, United Kingdom; ^3^Mass Spectrometry Centre, Centro di Servizi di Spettrometria di Massa, Università degli Studi di Firenze, Florence, Italy

**Keywords:** *Apis mellifera*, nurses, guards, foragers, queens, olfaction, odorant-binding proteins, chemosensory proteins

## Abstract

Reproductive and task partitioning in large colonies of social insects suggest that colony members belonging to different castes or performing different tasks during their life (polyethism) may produce specific semiochemicals and be differently sensitive to the variety of pheromones involved in intraspecific chemical communication. The main peripheral olfactory organs are the antennal chemosensilla, where the early olfactory processes take place. At this stage, members of two different families of soluble chemosensory proteins [odorant-binding proteins (OBPs) and chemosensory proteins (CSPs)] show a remarkable affinity for different odorants and act as carriers while a further family, the Niemann-Pick type C2 proteins (NPC2) may have a similar function, although this has not been fully demonstrated. Sensillar lymph also contains Odorant degrading enzymes (ODEs) which are involved in inactivation through degradation of the chemical signals, once the message is conveyed. Despite their importance in chemical communication, little is known about how proteins involved in peripheral olfaction and, more generally antennal proteins, differ in honeybees of different caste, task and age. Here, we investigate for the first time, using a shotgun proteomic approach, the antennal profile of honeybees of different castes (queens and workers) and workers performing different tasks (nurses, guards, and foragers) by controlling for the potential confounding effect of age. Regarding olfactory proteins, major differences were observed between queens and workers, some of which were found to be more abundant in queens (OBP3, OBP18, and NPC2-1) and others to be more abundant in workers (OBP15, OBP21, CSP1, and CSP3); while between workers performing different tasks, OBP14 was more abundant in nurses with respect to guards and foragers. Apart from proteins involved in olfaction, we have found that the antennal proteomes are mainly characterized by castes and tasks, while age has no effect on antennal protein profile. Among the main differences, the strong decrease in vitellogenins found in guards and foragers is not associated with age.

## Introduction

Colony organization and task partitioning in social insects largely depends on chemical communication, particularly in large communities. Pheromones regulate several aspects of social life (Leonhardt et al., 2016), such as hierarchy, reproduction control, recruitment to foraging sites, brood care, colony defense, nestmate recognition, and mate search. Moreover, sensitivity to food source odors, such as floral volatiles in bees, is fundamental for efficient foraging (Raguso, 2008).

Reproductive and sterile females perform different tasks: queens or queen-like individuals hardly leave the nest, while a large number of workers perform their tasks outside the nest, foraging being the main one. Moreover, an additional specialization can occur within the worker caste, with individuals performing different tasks during their life, as in honeybees, or being in some species both behaviorally and morphologically specialized. In most eusocial insects, caste and task differentiation may lead females to work for large parts of their life in different environments where specific sensory abilities are required. Moreover, individuals may interact with nestmates of different castes and ages (for instance with reproductive individuals or immature brood), thus being exposed to semiochemicals of different chemical nature.

The large repertoire of compounds secreted by pheromonal glands in social insects, together with the variety of volatiles present in the environment need to be analyzed by an efficient olfactory system (Wittwer et al., 2017). Differences in the perception of environmental and conspecific odorants between castes or during the life cycle has so far received limited attention compared to other aspects of phenotype plasticity (Kelber et al., 2010; Zhang et al., 2016; Nie et al., 2018).

Among social hymenopterans, the European honeybee, *Apis mellifera*, was the first species in which olfaction was studied at the molecular and neurophysiological levels (Deisig et al., 2006; Forêt and Maleszka, 2006; Robertson and Wanner, 2006; Forêt et al., 2007). This fact, together with the good knowledge on the chemical nature of pheromones involved both in colony communication and sexual behavior (Bortolotti and Costa, 2014) make the honeybee a model organism for the study of chemical communication and olfaction in insects.

Antennal chemosensilla are the main peripheral olfactory organs, where uptake, binding, transport, signal transduction, and signal inactivation occur. Odorants enter through cuticular pores, cross the sensillar lymph and reach the membrane of olfactory neurons (ONs), where two classes of receptors, ORs (olfactory receptors) and IRs (ionotropic receptors) are expressed.

Within the chemosensilla, the dendrites of ONs are bathed in the sensillar lymph containing high concentrations of small soluble proteins, carriers for odorants and pheromones (Pelosi et al., 2006, 2014, 2018; Leal, 2013). Three classes of these proteins have been described so far, but also in other organs producing pheromones. In fact, dual roles have been demonstrated for several members of these proteins, in detecting and releasing semiochemicals (Pelosi et al., 2018). Odorant-binding proteins (OBPs) were the first to be discovered (Vogt and Riddiford, 1981) and currently are the best studied group of olfactory carrier proteins both at the structural level, with more than 20 three-dimensional structures solved (Tegoni et al., 2004), four of which in the honey bee (OBP1: Pesenti et al., 2008; OBP2: Lescop et al., 2001; OBP5: unpublished, PDB: 3R72; OBP14: Spinelli et al., 2012), and at functional level (Pelosi et al., 2006). OBPs are 120–150 amino acid long, present a compact structure made of six α-helical domains and reversibly bind odorants and pheromones with micromolar dissociation constants (Pelosi et al., 2006). Several pieces of evidence have shown that their presence is important for a correct detection of chemical stimuli (Xu et al., 2005; Grosse-Wilde et al., 2006; Forstner et al., 2009; Swarup et al., 2011; Sun et al., 2012; Shiao et al., 2015; Zhang et al., 2017).

Chemosensory proteins (CSPs) is the second class of carrier proteins, smaller than OBPs (110–130 amino acids), also made in α-helical segments, but folded in structures different from those of OBPs (Pelosi et al., 2006). Three CSP structures have been solved (Lartigue et al., 2002; Campanacci et al., 2003; Tomaselli et al., 2006; Jansen et al., 2007) but none belong to honeybee. Like OBPs, several CSPs have been studied at the functional level and show to bind both general odorants and pheromones (Pelosi et al., 2014, 2018).

The third class of insect carrier proteins, NPC2 (Niemann-Pick type C2 protein) has been studied only recently. Although NPC2 proteins have been known for a long time in vertebrates as cholesterol carriers (Storch and Xu, 2009), it was only in the last few years that these proteins were proposed as semiochemical carriers in arthropods, mainly based on their large duplication and differentiation in this phylum (Pelosi et al., 2014). Their localization in chemosensilla and their affinity to small volatile molecules provided further support to this hypothesis (Ishida et al., 2014; Iovinella et al., 2016; Zhu et al., 2018). NPC2 proteins present a folding similar to lipocalins (Flower et al., 2000), with eight β-sheets assembled in a sort of compact β-barrel (Xu et al., 2007).

The genome of the honeybee contains 21 genes encoding OBPs, 6 encoding CSPs, and 5 encoding NPC2. Proteomic studies have identified 13 OBPs, 2 CSPs, and 2 NPC2 in the antennae of workers (Dani et al., 2010; Chan et al., 2013). Some of these proteins and others of the same families are also abundantly expressed in mandibular glands, where they likely assist release of pheromones, with expression patterns related to caste and age (Iovinella et al., 2011).

Previous work has demonstrated that whole-body, haemolymph, and brain protein profiles differ between honeybee queens and workers as well as between hive workers (i.e., workers performing activities inside the nest) and foragers (Engels and Fahrenhorst, 1974; Hummon et al., 2006; Corona et al., 2007; Wolschin and Amdam, 2007a,b; Garcia et al., 2009; Hernández et al., 2012). It has been proposed that the proteomic divergence might reflect the different life history of the two castes and, within workers, be partially explained by a shift in physiological and metabolic requirements as individuals approach different tasks (Corona et al., 2007; Wolschin and Amdam, 2007b; Garcia et al., 2009).

**FIGURE 1:**
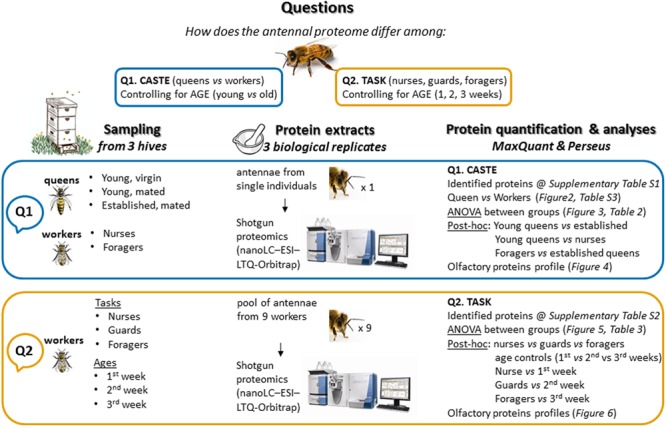
Scheme of the overall study protocol.

Here, we provide a comprehensive characterization of the antennal proteome of *Apis mellifera* in a functional perspective, through a shotgun proteomic approach. We address the question of how protein expression, both in general and with particular reference to soluble olfactory proteins, is related to castes, to different tasks of workers and to ages (**Figure 1**). We show that antennal protein profile, besides changing according to castes, also differs between workers performing different tasks, while it does not appear to be shaped by age.

## Materials and Methods

The overall study protocol is shown in **Figure 1**.

### *Apis mellifera* Rearing and Sampling

All specimens of *Apis mellifera ligustica* originated from hives housed at the Department of Biology of the University of Florence (Florence, Central Italy).

Queens of three physiological stages (virgin, newly mated, and established) and workers (i.e., nurses and foragers) originated from three different hives.

First and second instar larvae from three different colonies were reared into queens by transferring them into plastic queen cell cups which were inserted into orphanised colonies maintained within Apidea mating hives. Queens aged 2–4 days were collected either before (virgin, *n* = 3) or after mating flights (newly mated, *n* = 3). Fertile queens aged about 1 year (established queens, *n* = 3) were removed from the same colonies from which also nurses (*n* = 3) and foragers (*n* = 3) were collected. Nurses were identified by inspecting brood combs of each hive and searching for bees repeatedly attending brood cells, i.e., bees inserting their head and thorax in a cell containing a larva for at least 5 s (Withers et al., 1993; Crailsheim et al., 1996), while foragers were collected among bees returning from the foraging flights that gathered at the entrance of each hive after blocking it with a grid. All specimens were introduced into plastic tubes, transferred to the lab and soon killed by freezing.

Worker bees performing three different tasks (nurses, guards, and foragers) and of three different ages (1, 2, and 3 weeks) to be used as control, were collected from the same three hives.

Specific worker tasks might require a sensory specialization. Nurses inside the hive should be able to perceive queen and brood-specific semiochemicals emitted by the queen and larvae to respond to their requests (Bortolotti and Costa, 2014); guard bees at the hive entrance may specialize to recognize the difference in the chemical profile of conspecific approaching the colony in order to discriminate nestmates from potential intruders (Breed, 1998) as well as health from diseased individuals (Baracchi et al., 2012; Cappa et al., 2016), while foragers should be equipped to detect different floral odors identifying the flowering plants which provide the richest rewards.

Nurses and foragers were identified as described above. Bees were identified as guards if they patrolled the entrance board with their wings held open, chasing landing bees and inspecting or attacking other bees (Butler and Free, 1952; Downs and Ratnieks, 2000; Cappa et al., 2014, 2016).

Bees of different known ages were obtained by marking newly emerged workers for 5 weeks and collecting them at intervals of 7 days, so to obtain individuals aged 1, 2, and 3 weeks.

Combs with sealed brood, freed from adult individuals with a bee brush, were transferred to the nearby laboratory where workers emerging during the following 2 h were marked (using Uni Posca^®^paints). Combs and marked bees were then reinserted into their hives. Starting from the second up to the eighth week, marked workers of 1, 2, and 3 weeks were collected from the hives. Workers aged 1, 2, and 3 weeks were considered as control for, respectively, nurses, guards, and foragers (Moore et al., 1987; Breed et al., 1990, 2004; Withers et al., 1993; Crailsheim et al., 1996).

### Preparation of Proteins Samples and Analysis

Dissections were performed immediately before protein extractions and the following samples were prepared: antennae from single queens (virgin, mated, and established) and from single workers (nurses and foragers); pools of antennae from 9 workers (3 from each hive) performing different tasks (nurses, guards, and foragers) and of different age (1, 2, and 3 week-old,). Three biological replicates for each sample were prepared.

The extracts from collected samples were prepared by crushing the tissue in a mortar under liquid nitrogen and the proteins extracted with 6M Urea/2M Thiourea in Tris-Cl 50 mM pH 7.4. The protein extracts were centrifuged at 14.000 rpm for 40 min at 4°C and the supernatants were collected for the analysis. The total amount of protein in each sample was assessed by the Bradford colorimetric assay (Bradford, 1976), with the “Bio-Rad Protein Assay” kit using serial dilutions of bovine serum albumin to generate a standard curve. Protein sample concentration was measured by Infinite PRO 200 reader (TECAN).

Protein extract were prepared, processed and analyzed on a nanoLC-ESI-LTQ-Orbitrap mass spectrometer as described in Iovinella et al. (2015).

### Reagents

Ammonium bicarbonate, DTT, iodoacetamide, sodium chloride, formic acid, acetonitrile, trifluoroacetic acid, acetic acid, and thiourea were from Sigma-Aldrich (Milan, Italy), while Tris and urea from Euroclone. Trypsin was purchased from Promega (Sequencing Grade Modified Trypsin) and Lys-C from Thermo Scientific (MS grade). The hand-made desalting/purification STAGE column were prepared using three C18 Empore Extraction Disks (3M).

### Protein Identification and Quantification

The identification of proteins was performed using MaxQuant software (version 1.5.2.6) (Cox and Mann, 2008). The derived peak list was searched with Andromeda search engine (Cox et al., 2011). We used as database all the proteins of *Apis mellifera* from Uniprot merged with a set of commonly observed contaminants, such as human keratins, bovine serum proteins, and proteases. Additional variable modifications were set for sequences of antimicrobial peptides (sequences downloaded from Uniprot^1^) in ‘Group-specific parameters.’ In the parameter section, we set as enzyme Trypsin and Lys-C, allowing up to two missed cleavages. The minimum required peptide length was seven amino acids. Carbamidomethylation of cysteine and oxidation of methionine were set as variable modifications. As no labeling was performed, multiplicity was set to 1. During the main search, parent masses were allowed an initial mass deviation of 4.5 ppm and fragment ions were allowed a mass deviation of 0.5 Da. PSM (peptide spectrum match) and protein identifications were filtered using a target-decoy approach at a false discovery rate (FDR) of 1%.

Relative, label-free quantification (LFQ) of proteins was done using the MaxLFQ algorithm integrated into MaxQuant. The match between runs option was enabled with a match time window of 2 min and an alignment time window of 20 min. For protein quantification we used 1 as minimum ratio count, “Unique+Razor” peptides (i.e., those exclusively shared by the proteins of the same group), peptides with variable modifications, and selected “discard unmodified counterpart peptide.”

### Data Analysis

The data relative to identification and quantification are contained in the MaxQuant output files named proteinGroups.txt and are reported in **Supplementary Table S1** for the queens and control workers, and **Supplementary Table S2** for workers of different age and task. Acquisition methods, databases used, and raw files are available through ProteomeXchange^2^ (accession: PXD009062).

Further analysis of the MaxQuant-processed data was performed using Perseus software (version 1.5.1.6). Annotations according to gene ontology (GO) categories, Protein family (Pfam) and InterPro were downloaded from the link available in Perseus software^3^ and each protein identifier was associated with those categories if available. The data were filtered to eliminate hits to the reverse database, contaminants and proteins only identified with modified peptides.

Differences in single protein levels were first evaluated between queens and workers. A Venn diagram was drawn between queens (virgin, mated, and established) and workers (nurse and foragers), considering “Unique+Razor” peptides identified in at least 3 replicates, out of 9 for queens, and 2 replicates, out of 6, for workers. Differences in single protein levels were evaluated between the two castes, independently from age and/or physiological stage, considering only proteins with at least 5 observations (out of 15), through a *t*-test on log_2_ transformed LFQ intensity values, with a FDR = 0.05 (permutation based false discovery rate), number of randomization set to 1000 and S0 set to 0.1. This latter value is an artificial within groups variance which controls both the relative importance of *t*-test *p*-value and difference between means (Tusher et al., 2001). Differential expression analysis between queens and workers of different ages and/or physiological stages was performed using ANOVA, where *p*-values were Benjamini Hochberg corrected at 5% FDR. A *post hoc* two-sample *t*-test, with the same correction, was applied to determine differences in single protein levels between antennae of workers and queens, compared according age, and between queens at different physiological stages. Hierarchical clustering analyses were performed using average Euclidean distance and the default parameters of Perseus (300 clusters, maximum 10 iterations).

The same approach was used to evaluate differences between workers of different tasks and ages. Differential expression analysis was performed using ANOVA, where *p*-values were Benjamini Hochberg corrected at 5% FDR, considering only proteins with at least 6 observations (out of 18). A *post hoc* two-sample *t*-test, with the same correction, was applied to determine differences in single protein levels between antennae of workers performing different tasks, as well as comparing them with the respective age control samples. Hierarchical clustering analyses were performed using average Euclidean distance and the default parameters of Perseus (300 clusters, maximum 10 iterations).

Differential expression of olfactory proteins (OBPs, CSPs, NPC2, and ORs) and odorant degrading enzymes (ODEs) was further analyzed by considering reduced datasets containing only data of these proteins. Missing LFQ values were imputed (width = 0.3, downshift = 1.8), and 0 was manually substituted when values were missing in all replicates of one caste/task/age category. *T*-test (Benjamini Hochberg corrected at 5% FDR) was calculated on these data.

## Results and Discussion

Aim of this work was a proteomic analysis of antennae of honeybees belonging to different castes (queens and workers) and of workers performing different tasks; for these latter bees of known ages were used as control, in order to understand if age influences protein expression profile.

### Differences Between Castes

Search of LC-MS data acquired for antennal extracts from single individuals (queens and control workers) identified 395 proteins. Data regarding the identification of all proteins, together with other information (accessions, scores, percent coverage, missed cleavages, etc.) are reported in **Supplementary Table S1**.

Firstly, we compared the global expression of proteins between the two castes, regardless of age and/or physiological stage (nurses and foragers as workers vs. young virgin, young mated, and established queens).

We obtained a comparable distribution of protein families, with the PBP/GOBP family as the most represented in both castes. Thirteen proteins were exclusively found in queens; none of these proteins have been reported to have a role in olfaction or be linked to caste differentiation (**Table 1**), except for Major royal jelly protein 1 (acc. O18330), that is the most abundant protein found in the royal jelly, the food of the queen honey bee larva that determines the development of the young larvae and is responsible for the high reproductive ability of honeybee queens (Buttstedt et al., 2014).

**Table 1:** Proteins exclusively found in queens.

Protein IDs	Description	Pfam	Razor + unique peptides	Sequence coverage [%]	Mol. weight [kDa]
A0A087EP48	Ribosome-recycling factor	RRF	1	7	20.483
A0A087ZNF8	Gamma-interferon-inducible-lysosomal thiol reductase		1	5.2	25.5
A0A087ZPK0	Lambda crystallin-like protein	3HCDH	1	3.8	35.86
A0A087ZSH6	Derlin-2		1	2.5	27.939
A0A087ZVX3	Glucose dehydrogenase	GMC_oxred_C	1	1.9	70.028
Q25BT6	Alpha-glucosidase	Alpha-amylase	2	4.6	65.578
A0A088A4U6	Cuticular protein 17 precursor	Chitin_bind_4	2	13.9	17.47
A0A088AAT2	Venom serine protease 34	Trypsin	3	9.1	42.137
A0A088AB75	Uncharacterized protein	LRR_8	11	34.1	50.285
A0A088AC16	Uncharacterized protein		1	8.8	19.433
A0A088AEW2	Uncharacterized protein	Kazal_1	1	6.9	14.484
A0A088ASZ6	Transgelin	Calponin	2	15.2	20.464
O18330	Major royal jelly protein 1	MRJP	9	27.4	46.86

**FIGURE 2:**
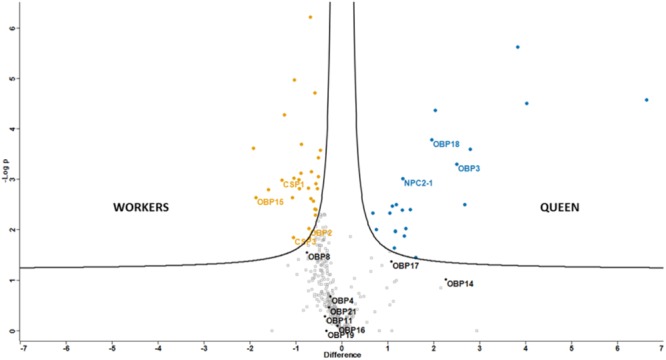
Graphical representation (Volcano plot) of differential protein expression between castes. Proteins significant to *t*-test (FDR = 0.05) are reported in orange, for workers, and blue, for queens. Soluble olfactory proteins are labeled with their names.

Abundance (log_2_ transformed LFQ values) of proteins quantified in at least 5 out of the 15 samples, was compared through a *t*-test (FDR = 0.05) and graphically represented by a volcano plot (**Figure 2**); 20 and 31 proteins were more expressed in workers and queens, respectively (**Supplementary Table S3**). In workers two OBPs (OBP2 and OBP15) and two CSPs (CSP1 and CSP3) were significantly more expressed, together with several enzymes possibly involved in degradation of odors and/or pheromones, a couple of structural proteins (calreticulin and tubulin) and enzymes involved in various biological processes, such as metabolism and transport.

Among proteins significantly more abundant in queens there are two OBPs (OBP3 and OBP18) and the NPC2-1, two cuticle proteins and several lipid transport proteins, among which we found two apolipophorins and three vitellogenins. Differences of olfactory proteins ranged from 2 (OBP2, CSP3, and NPC2-1) to around 4 times.

The data regarding olfactory proteins are in good agreement with those reported by Chan et al. (2013), where a proteomic study of different organs of *Apis mellifera* belonging to different castes was conducted; quantitative differences between queens and workers were comparable, apart from OBP15, which was not found in their work.

**FIGURE 3:**
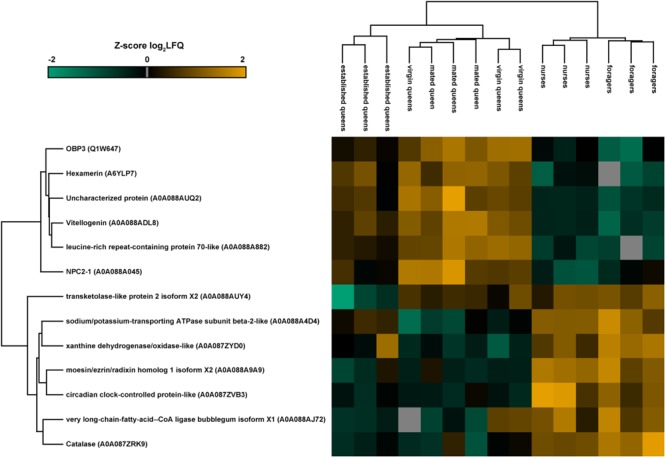
Heatmap representation of the expression of proteins significantly different (one-way ANOVA, Benjamini Hochberg-corrected FDR = 5%) between groups of both castes. The map has been built making an unsupervised hierarchical clustering (300 clusters, maximum 10 iterations) based on LFQ (label-free quantification). Uniprot accession numbers are reported in brackets. Color scale reports *Z*-score log2 transformed LFQ intensity values. Missing data are reported in gray. Groups belonging to the two castes are clearly separated, as displayed in the cluster grouping biological replicates.

Quantitative differences in protein expression between the single groups (nurse, foragers, virgin queens, mated queens, and established queens) belonging to the different castes were evaluated through one-way ANOVA (Benjamini Hochberg-corrected FDR = 5%). The heatmap reported in **Figure 3** shows the 13 proteins differentially expressed between castes (**Table 2**). Most of them are ‘uncharacterized proteins’; about one half present a higher expression in queens, including the OBP3 and two storage proteins (a vitellogenin and a hexamerin). This latter finding reflects their physiological role. In fact, vitellogenin has been reported to act as an antioxidant to promote longevity in queen bees (Corona et al., 2007). The presence of the hemolymph protein hexamerin 70 in the antennae has been reported in young queens (4 days old) and it has been suggested that it could be used in the building up of antennal cuticle structures and it could be related to modifications of the external structure of the sensilla placodea (Danty et al., 1998).

**Table 2:** Proteins differentially expressed in single groups of the two castes, according to one-way ANOVA (Benjamini Hochberg-corrected FDR = 5%) and to *post hoc* two-sample Student’s *t*-tests.

Uniprot accession number	Description	Pfam	–Log ANOVA *p*-value	ANOVA *q*-value	Comparison	–Log Student’s *t*-test *p*-value	Student’s *t*-test Test statistic
A0A087ZRK9	Catalase	Catalase	3.37	0.03	Nurse-young queen;	2.21;	3.86;
					Foragers-established queen	2.81	7.67
A0A087ZVB3	Circadian clock- controlled protein-like	JHBP	3.00	0.04	Nurse-young queen;	3.02;	5.46;
					Foragers-established queen	2.01	4.64
A0A087ZYD0	Xanthine dehydrogenase/oxidase-like	Ald_Xan_dh_C	2.98	0.03	Nurse-young queen	3.13	5.68
A0A088A045	NPC2-1	E1_DerP2_DerF2	3.16	0.04	Nurse-young queen	3.21	–5.87
A0A088A4D4	Sodium/potassium-transporting ATPase subunit beta-2-like	Na_K-ATPase	3.04	0.04	Nurse-young queen	3.00	5.42
A0A088A882	Leucine-rich repeat- containing protein 70-like	LRR_8	4.83	0.003	Nurse-young queen;	4.36;	–8.95;
					Foragers-established queen;	2.83;	–11.30;
					Young-established queen	2.99	5.38
A0A088A9A9	Moesin/ezrin/radixin homolog 1 isoform X2	ERM	3.71	0.02	Nurse-young queen;	4.66;	9.96;
					Foragers-established queen	1.7	3.75
A0A088ADL8	Vitellogenin	DUF1943	5.19	0.00	Nurse-young queen;	4.45;	–9.27;
					Foragers-established queen;	2.28;	–5.51;
					Young-established queen	1.88	3.29
A0A088AJ72	Very long-chain-fatty-acid–CoA ligase bubblegum isoform X1	AMP-binding	3.08	0.04	Foragers-established queen	2.32	5.68
A0A088AUQ2	Uncharacterized protein		3.64	0.02	Nurse-young queen;	3.11;	–5.64;
					Foragers-established queen	1.85	–4.17
A0A088AUY4	Transketolase-like protein 2 isoform X2	Transket_pyr	2.84	0.04	Foragers-established queen;	1.93;	4.39;
					Young-established queen	2.67	4.73
A6YLP7	Hexamerin	Hemocyanin_C	2.90	0.04	Nurse-young queen;	3.06;	–5.54;
					Foragers-established queen	1.53	–3.92
Q1W647	OBP3	PBP_GOBP	3.49	0.02	Nurse-young queen;	3.77;	–7.26;
					Young-established queen	2.99	5.39

Protein profiles of queens of different age/stage were compared among them and toward those of the corresponding group of workers through a *t*-test (Benjamini Hochberg-corrected FDR = 2%).

No proteins differed between virgin and mated queens, therefore, we pooled the two groups together as young queens and we compared them with nurses (workers of comparable age) and established queens (older queens in a different physiological stage). Since several differences were observed with respect to established queens, we can deduce that a stable reproductive status also affects antennal protein expression pattern, while mating has little or no effect on it.

The highest number of differences in protein abundance was obtained comparing nurses with virgin and mated queens, showing that NPC2-1, OBP3, Vitellogenin (acc. A0A088ADL8), and Hexamerin (acc. A6YLP7) are typical of young queens, while the circadian clock-controlled protein-like, a Haemolymph juvenile hormone (JH) binding protein, characterizes both nurses and foragers compared to young queens and established queens, respectively. The *t*-test statistics concerning each comparison are reported in **Table 2**.

**FIGURE 4:**
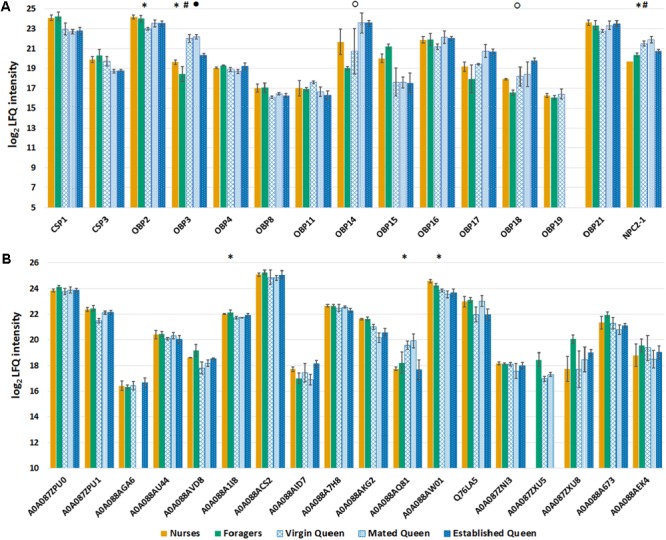
**(A)** Bar chart reporting the log_2_ transformed and imputed LFQ intensity values of the olfactory proteins, averaged for biological replicates (±SE). Proteins marked with a symbol are significant to *t*-test (Benjamini Hochberg-corrected FDR = 5%) for the comparison nurse-virgin queen (asterisk), nurse-mated queen (hash), mated-established queen (filled circle), and foragers-established queen (circle). **(B)** Bar chart reporting the log_2_ transformed and imputed LFQ intensity values of the odorant degrading enzymes (ODEs), indicated with Uniprot accession number, averaged for biological replicates (±SE). Protein marked with an asterisk are significant to *t*-test (Benjamini Hochberg-corrected FDR = 5%) between nurse and young queens (virgin and mated).

Besides the global expression pattern of antennal proteins, our primary aim was to analyze how castes influence the profile of olfactory proteins. We identified 12 of the 21 predicted OBPs, 2 of the 6 predicted CSPs, and 1 out of the 5 NPC2, in our proteomic analysis and we have analyzed their expression comparing queens and workers of the same age (**Figure 4A**). The *t*-tests (Benjamini Hochberg-corrected FDR = 5%) performed considering only these proteins showed that, in the comparison between nurse and virgin queens, OBP2 is significantly more abundant in nurse, while OBP3 and NPC2-1 are more expressed in queens. Moreover, OBP3 is more abundant in mated compared to established queens, confirming that this protein characterizes young queens. In the comparison between foragers and established queens the proteins OBP14 and OBP18 were more abundant in established queens. OBP2 has been found to have a good affinity for components (2-heptanone, isoamyl acetate) of alarm pheromone (Briand et al., 2001), while OBP3 binds benzoate (Dani et al., 2010), although information is not available for methyl p-hydroxybenzoate, one of the major components of the queen mandibular gland. OBP18, together with OBP16, has been reported to be more expressed in workers with higher hygienicity and bind long chain fatty acids and their ethyl and methyl esters (Guarna et al., 2015), some of which are constituents of the brood pheromone.

In addition to OBPs, CSPs, and NPC2, other protein families are involved in peripherical processes of odor perception in insects, in particular the ODEs, involved in inactivation through degradation of the chemical signals, once the message is conveyed. Among the Pfams containing proteins that have been reported to be involved in this process (Yu et al., 2009; Durand et al., 2012; Leal, 2013), we selected those significantly enriched (Fisher exact test; Benjamini Hochberg-corrected FDR = 0.02) and we evaluated differences between single groups of both castes (**Figure 4B**). Only in the comparison between nurses and young queens three proteins were statistically significant (*t*-test): the delta-1-pyrroline-5-carboxylate dehydrogenase, mitochondrial (acc. A0A088A1I8), and the esterase FE4-like (acc. A0A088AW01) more expressed in nurses, while the esterase E4-like (acc. A0A088AQ81) is significantly more abundant in young queens. The *t*-test statistics for olfactory proteins, concerning each comparison, are reported in **Supplementary Table S4**.

### Differences Between Tasks

To understand which factor, different tasks and/or age, could influence protein expression in workers we analyzed antennae from pools of nurses, guards, and foragers (different tasks) and of workers of comparable age (1, 2, or 3 weeks, respectively), but for which specific task was not assessed. Search of LC-MS data acquired for pools of antennae of workers identified 530 proteins. Data regarding the identification of all proteins, together with other information (accessions, scores, percent coverage, missed cleavages, etc.) are reported in **Supplementary Table S2**.

**FIGURE 5:**
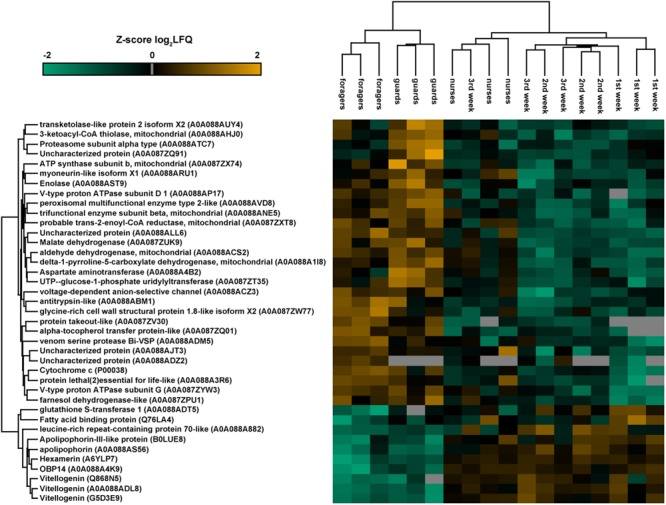
Heatmap representation of the expression of proteins significantly different (one-way ANOVA, Benjamini Hochberg-corrected FDR = 5%) between groups of workers of different tasks and ages. The map has been built making an unsupervised hierarchical clustering (300 clusters, maximum 10 iterations) based on LFQ (Label-free quantification). Uniprot accession numbers are reported in brackets. Color scale reports *Z*-score log2 transformed LFQ intensity values. Missing data are reported in gray. Major differences are between old workers (guards and foragers) and nurse, which are in the same cluster of bees with undetermined task.

Considering separately each group of workers of different tasks and ages, we did not find proteins exclusively expressed in one group. Differences in protein expression between groups of different tasks (nurse, guards, and foragers) and ages (first, second, and third week) were evaluated through one-way ANOVA on log_2_ transformed LFQ values (Benjamini Hochberg-corrected FDR = 5%). The heatmap reported in **Figure 5** shows the 39 proteins differentially expressed between castes (**Table 3**). Most of them (29 proteins) are enzymes and present higher expression in guards and foragers, with respect to nurse, which are closer to workers of know age for which task was not assessed.

**Table 3:** Proteins differentially expressed in single tasks/ages groups of workers, according to one-way ANOVA (Benjamini Hochberg-corrected FDR = 5%) and to *post hoc* two-sample Student’s *t*-tests.

Protein IDs	Descriptor	Pfam	–Log ANOVA *p*-value	ANOVA *q*-value	Comparison	–Log Student’s *t*-test *p*-value	Student’s *t*-test Test statistic
G5D3E9	Vitellogenin	Vitellogenin_N	4.32	0.01	Nurses-guards;	2.46;	6.20;
					Nurses-foragers;	2.9;	8.11;
					Guards-2^nd^ week;	2.12;	–4.98;
					Foragers-3^rd^ week	2.15	–5.07
A0A087ZPU1	Farnesol dehydrogenase-like	adh_short	3.49	0.02	guards-2^nd^ week	1.67	3.66
A0A087ZQ01	Alpha-tocopherol	CRAL_TRIO	4.04	0.01	Nurses-guards;	2.58;	–6.65;
	transfer protein-like				Nurses-foragers;	2.52;	–6.42;
					Guards-2^nd^ week;	2.01;	4.65;
					Foragers-3^rd^ week	1.71	3.77
A0A087ZQ91	Uncharacterized protein	AAA_8	3.36	0.02	Guards-2^nd^ week	1.85	4.16
A0A087ZT35	UTP–glucose-1-phosphate uridylyltransferase	UDPGP	2.57	0.04	guards-2^nd^ week	2.34	5.75
A0A087ZUK9	Malate dehydrogenase	Ldh_1_C	2.46	0.05	guards-2^nd^ week	2.04	4.73
A0A087ZV30	Protein takeout-like	JHBP	3.31	0.02	Guards-2^nd^ week;	1.56;	3.39;
					Foragers-3^rd^ week	3.33	10.51
A0A087ZW77	Glycine-rich cell wall structural protein 1.8-like isoform X2		3.04	0.02	Foragers-3^rd^ week	2.73	7.31
A0A087ZX74	ATP synthase subunit b, mitochondrial	Mt_ATP-synt_B	3.08	0.02	Guards-2^nd^ week	2.06	4.79
A0A087ZXT8	Probable trans-2-enoyl-CoA reductase, mitochondrial	ADH_N	3.78	0.01	Guards-2^nd^ week;	2.74;	7.35;
					Foragers-3^rd^ week	1.86	4.20
A0A087ZYW3	V-type proton ATPase subunit G		3.46	0.02			
A0A088A1I8	Delta-1-pyrroline-5-carboxylate dehydrogenase, mitochondrial	Aldedh	3.08	0.02	guards-2^nd^ week	2.25	5.42
A0A088A3R6	Protein lethal(2)essential for life-like	HSP20	2.92	0.03	Guards-2^nd^ week;	2.18;	5.19;
					Foragers-3^rd^ week	3.16	9.46
A0A088A4B2	Aspartate aminotransferase	Aminotran_1_2	2.61	0.04	Guards-2^nd^ week	1.74	3.85
A0A088A4K9	OBP14	PBP_GOBP	4.70	0.01	Nurses-guards;	2.35;	5.78;
					Nurses-foragers;	2.36;	5.81;
					Guards-2^nd^ week;	1.78;	–3.97;
					Foragers-3^rd^ week	2.2	–5.25
A0A088A882	Leucine-rich repeat-containing protein 70-like	LRR_8	3.28	0.02	Guards-2^nd^ week;	3.2;	–9.71;
					Foragers-3^rd^ week	1.82	–4.08
A0A088ABM1	Antitrypsin-like	CTDII	2.83	0.03	Nurses-foragers;	2.69;	–7.13;
					Foragers-3^rd^ week	2.36	5.83
A0A088ACS2	Aldehyde dehydrogenase, mitochondrial	Aldedh	3.24	0.02	Guards-2^nd^ week	2.12	4.99
A0A088ACZ3	Voltage-dependent anion-selective channel	Porin_3	2.54	0.04	Guards-2^nd^ week	1.75	3.89
A0A088ADL8	Vitellogenin	DUF1943	5.04	0.005	Nurses-foragers;	2.95;	8.36;
					Guards-2^nd^ week;	2.36;	–5.8;
					Foragers-3^rd^ week	2.85	–7.87
A0A088ADM5	Venom serine protease Bi-VSP	CLIP	2.52	0.04	Guards-2^nd^ week;	1.57;	3.42;
					Foragers-3^rd^ week	1.77	3.95
A0A088ADT5	Glutathione S-transferase 1	GST_N	2.87	0.03	Foragers-3^rd^ week	2.02	–4.67
A0A088ADZ2	Uncharacterized protein		2.74	0.03			
A0A088AHJ0	3-ketoacyl-CoA thiolase, mitochondrial	Thiolase_C	2.75	0.03	Guards-2^nd^ week	3.00	8.63
A0A088AJT3	Uncharacterized protein	IATP	2.46	0.05	Guards-2^nd^ week;	1.49;	3.21;
					Foragers-3^rd^ week	2.33	5.71
A0A088ALL6	Uncharacterized protein	Vitellogenin_N	2.96	0.03	Guards-2^nd^ week	3.48	11.48
A0A088ANE5	Trifunctional enzyme subunit beta, mitochondrial	Thiolase_C	2.93	0.03	Guards-2^nd^ week	2.25	5.40
A0A088AP17	V-type proton ATPase subunit D 1	ATP-synt_D	3.33	0.02	Guards-2^nd^ week	2.73	7.30
A0A088ARU1	Myoneurin-like isoform X1		2.87	0.03	Guards-2^nd^ week	2.22	5.31
A0A088AS56	Apolipophorin	DUF1081	4.12	0.01	Nurses-guards;	2.84;	7.81;
					Guards-2^nd^ week	4.18	–17.28
A0A088AST9	Enolase	Enolase_C	2.46	0.05	Guards-2^nd^ week	1.71	3.77
A0A088ATC7	Proteasome subunit alpha type	Proteasome	3.49	0.02	Nurses-guards;	2.08;	–4.86;
					Guards-2^nd^ week	2.7	7.19
A0A088AUY4	Transketolase-like protein 2 isoform X2	Transket_pyr	2.49	0.05	Guards-2^nd^ week	1.85	4.16
A0A088AVD8	Peroxisomal multifunctional enzyme type 2-like	adh_short	2.59	0.04	Nurses-guards;	2.07;	–4.82;
					Guards-2^nd^ week	2.66	6.99
A6YLP7	Hexamerin	Hemocyanin_C	4.67	0.004	Nurses-guards;	3.68;	12.90;
					Guards-2^nd^ week;	4.43;	–19.96;
					Foragers-3^rd^ week	1.84	–4.12
B0LUE8	Apolipophorin-III-like protein	ApoLp-III	3.12	0.02	Guards-2^nd^ week	2.10	–4.91
P00038	Cytochrome c	Cytochrom_C	4.06	0.01	Nurses-foragers;	2.16;	–5.11;
					Foragers-3^rd^ week	2.74	7.35
Q76LA4	Fatty acid binding protein	Ald_Xan_dh_C	2.49	0.05			
Q868N5	Vitellogenin	DUF1943	2.78	0.03	Nurses-foragers;	3.75;	13.44;
					Foragers-3^rd^ week	3.16	–9.5

Protein profiles of workers carrying out different tasks were compared to those of the corresponding coetaneous workers through a *t*-test (Benjamini Hochberg-corrected FDR = 5%). No differences were obtained comparing honeybees with defined ages (1st week versus 2nd and 3rd week, 2nd week versus 3rd week), as well as between guards compared to foragers and nurse compared to honeybees of 1st week. Major differences were obtained between guards compared to honeybees of 2nd week. Thus, the observed differences appear to be linked to the specific task performed by workers rather than by the different age. As already reported by previous studies, task specialization is often followed by biochemical and physiological specialization of bee workers (Robinson, 1987; Huang et al., 1994; Pearce et al., 2001; Amdam et al., 2003; Münch et al., 2008). Young nurses performing inside hive duties present high titers of vitellogenin (Huang et al., 1994; Amdam et al., 2003; Münch et al., 2008), whereas middle-aged guard bees and older foragers show very high levels of JH promoting, respectively, aggressive behavior in guards (Breed, 1983; Pearce et al., 2001) and the onset of foraging in older bees (Robinson, 1985, 1987; Sullivan et al., 2000; Elekonich et al., 2001).

In our samples, two vitellogenins, one hexamerin, and the OBP14 are more abundant in nurses and workers of 2nd and 3rd week with respect to guards and foragers, while a JH binding protein (acc. A0A087ZV30) is significantly more expressed in guards and foragers with respect to their age-control workers. The higher expression of a JH-binding protein may be linked to the higher titers of such hormone in these specific groups of workers (Breed, 1983; Robinson, 1985, 1987; Sullivan et al., 2000; Elekonich et al., 2001; Pearce et al., 2001). Among the enzymes, there are a ‘farnesol dehydrogenase-like’ protein (acc. A0A087ZPU1) and a ‘Cytochrome c’ (acc. P00038), whose function could be probably related to the inactivation of chemical signals, which are more expressed in guards and foragers, respectively. The *t*-test statistics concerning each comparison are reported in **Table 3**. The absence of conspicuous differences among workers of the different age groups (1st, 2nd, and 3rd week) compared to the ones observed in task-specific groups, may be due to the fact that each age groups is likely to include workers involved in different tasks. Indeed, variation in task performance among similarly aged workers is common in honeybee colonies (Winston, 1991; Huang et al., 1994) and the presence of bees performing different tasks in our age groups could mask the differences observed among task-specific groups.

A more detailed analysis was conducted on the expression patterns of soluble olfactory proteins, in order to understand if their profile could characterize workers of different tasks and ages.

**FIGURE 6:**
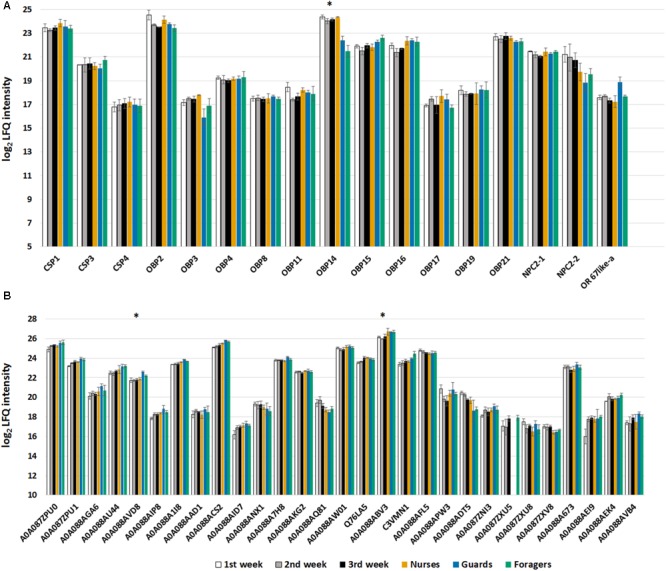
**(A)** Bar chart reporting the log_2_ transformed and imputed LFQ intensity values of the olfactory proteins, averaged for biological replicates (±SE). Proteins marked with an asterisk are significant to *t*-test (Benjamini Hochberg-corrected FDR = 5%) for the comparison nurse-old workers (guards and foragers). **(B)** Bar chart reporting the log2 transformed and imputed LFQ intensity values of the ODEs, indicated with Uniprot accession number, averaged for biological replicates (±SE). Proteins marked with an asterisk are significant to *t*-test (Benjamini Hochberg-corrected FDR = 5%) for the comparison 2nd week-guards.

We identified 11 of the 21 predicted OBPs, 3 of the 6 predicted CSPs, 2 out of 5 predicted NPC2, and one odorant receptor in our proteomic analysis (**Figure 6A**). We can observe that in this case, with respect to the samples from single individuals, we identified more proteins, and this is certainly due to the use of pools of antennae (from 9 bees). In fact, comparing nurses and foragers, for whom we have both single specimens and pooled samples, we observed an increase of 10% in the number of identified proteins. However, among OBPs, OBP18 was identified with only 1 peptide and the protein was included in the same protein group with OBP21 (**Figure 5**), while this was not the case in the single specimen samples. Moreover, in this analysis we found CSP4 and NPC2-2 that were not found in antennal extract from single individuals. Surprisingly we have also identified the odorant receptor 67a-like isoform X1 that being a transmembrane protein is not easy to solubilize given our mild protein extraction. In general, proteomic studies are more suitable to target soluble proteins than membrane proteins and our results are consistent with other similar analyses on insect chemosensory organs.

Unexpectedly, we did not find OBP1, that was instead identified in a 2D-gel spot of foragers antennae in our previous work, together with OBP16 (Dani et al., 2010). The expression of the OBP1 encoding gene was found to be limited to antennae and comparable between drones, queens, and foragers (Forêt and Maleszka, 2006); however, the protein is around 5 times more abundant in drones with respect to workers and queens (Chan et al., 2013), and this could explain why we don’t identify the protein in our sample. Apart from these differences, the abundances of all the OBPs and the CSPs identified in nurses and foragers of both datasets are strongly consistent.

The *t*-tests (Benjamini Hochberg-corrected FDR = 5%) performed only on olfactory proteins showed that, in the comparison between nurse and old workers (guards and foragers considered together) only the OBP14 was significantly more expressed (more than 2 times) in nurse, while none of the considered proteins was differentially expressed comparing workers with defined task and their age-controlled sample. The differences in OBP14 suggest that this protein can be involved in pheromonal communication within the hive rather than to perception of floral odors. This finds a biological correlation with the affinity for compounds reported for aggregation (farnesol, geraniol, and citral) or alarm pheromones (2-heptanone and isoamyl acetate) but not with the very strong affinity reported for eugenol (Iovinella et al., 2011).

Even in this case we selected Pfams significantly enriched (Fisher exact test; Benjamini Hochberg-corrected FDR = 0.02) containing proteins that have been reported as ODEs (**Figure 6B**). Differences have been detected only between guards and workers of 2nd week for a Glutathione S-transferase (acc. A0A088ABV3) and a ‘peroxisomal multifunctional enzyme type 2-like’ (acc. A0A088AVD8), which are both more expressed in guards. The *t*-test statistics for olfactory proteins are reported in **Supplementary Table S5**.

A similar approach to that used in the present work has been adopted for a comparative transcriptome analysis conducted on *Apis mellifera* antennae of workers performing different tasks by Nie et al. (2018). None of the proteins encoded by the genes reported as associated with nursing and foraging behavior were found to be differentially expressed in our samples. With regards to OBPs and CSPs, similarly to results by Nie et al. (2018), we also observed a decrease of OBP17 level from nursing to foraging task, although the difference in abundance was not statistically significant.

## Conclusion

This work presents for the first time a detailed proteomic investigation of *Apis mellifera* antennae where bees belonging to different castes, at different physiological stages, and workers performing different tasks have been compared. To control for age-related changes workers were also compared with bees of different ages but of unassessed task.

Expression analysis has highlighted differences between the two castes, including several proteins involved in olfaction. Among these, the NPC2-1 and the OBP3 characterize young and still not egg-laying queens, together with storage proteins well known for their role in caste determination (two vitellogenins and one hexamerin).

Major differences have been found between groups of workers performing different tasks and groups of defined age, while antennal protein profiles of honeybees at 1st, 2nd, and 3rd week do not show differences. Among the soluble olfactory proteins, we found that OBP14 is typical of nurse bees with respect to guards and foragers.

The data here reported are in good, although not complete, agreement with the results at the RNA level reported by Forêt et al. (2007) and the proteomic analysis of antennae between castes (Chan et al., 2013), while they have limited correspondence with the comparative transcriptomic work by Nie et al. (2018), where antennae of workers of different tasks were studied.

Our data suggest that caste, physiological stage and performed task shape the antennal profile of honeybees and that two OBPs and one NPC2 are differentially expressed. Since the binding properties have been defined only for a few honeybee soluble olfactory proteins, studies aimed at understanding how expression of these proteins associates with castes and with task transitions may suggest which semiochemicals should be targeted to clarify their physiological role.

## Ethics Statement

Honeybees used in this work were reared in semi-natural conditions. They were treated as well as possible given the constraints of the experimental design. This study was carried out in accordance with the Italian guidelines on animal wellness.

## Author Contributions

FD, RC, and ST designed the research. II, FC, AC, and IP performed the experiments. II and FD performed the statistical analysis and drafted the manuscript. All authors discussed the results during the progress of the work, participated in revising the article critically, helped finalizing the manuscript, and gave final approval for publication.

## Conflict of Interest Statement

The authors declare that the research was conducted in the absence of any commercial or financial relationships that could be construed as a potential conflict of interest.

## Footnotes

1 http://www.uniprot.org/uniprot/?query=antimicrobial+peptides+apis+mellifera&sort=score

2 www.proteomexchange.org

3 http://141.61.102.106:8080/share.cgi?ssid=0qF9uFn

## Supplementary Material

TABLE S1 Complete list of proteins identified in proteomic analysis of antennae from single individuals of nurses (N), foragers (F), virgin queens (RV), mated queens (RF), and established queens (Rold). The protein groups table contains information on the proteins identified in all processed raw-files. Each single row contains the group of proteins that could be reconstructed from a set of peptides.

TABLE S2 Complete list of proteins identified in proteomic analysis of antennae from pool of 9 individuals of nurses (N), guards (G), foragers (F) and workers aged 1-week (A), 2-week (B) and 3-week (C). The protein groups table contains information on the proteins identified in all processed raw-files. Each single row contains the group of proteins that could be reconstructed from a set of peptides.

TABLE S3 Proteins significantly different between castes (*t*-test, FDR = 0.05), graphically represented in volcano plot of **Figure 2**.

TABLE S4 Soluble olfactory proteins differentially expressed in single groups of the two castes (Student *t*-test Benjamini Hochberg-corrected FDR = 5%).

TABLE S5 Soluble olfactory proteins differentially expressed in groups of workers (Student *t*-test Benjamini Hochberg-corrected FDR = 5%).
