# Supplementary material for: Antennal Protein Profile in Honeybees: Caste and Task Matter More Than Age
Source: Front Physiol. 2018 Jun 20;9:748. doi: 10.3389/fphys.2018.00748 (PMC6019485; doi:10.3389/fphys.2018.00748)
Supplement: TABLE S3 — Proteins significantly different between castes (t-test, FDR = 0.05), graphically represented in volcano plot of Figure 2. [file Table_3.PDF]

Supplementary Table S3. Proteins significantly different between castes (t-test, FDR=0.05), graphically represented in volcano plot of Figure 2.

-LOG (P-value)	Difference	Protein IDs	Description	Pfam	Razor + unique peptides	Sequence coverage [%]	Mol. weight [kDa]
3.61	1.92	A0A088A3R5	protein lethal(2)essential for life-like	HSP20	4	28.4	22.56
2.63	1.87	Q1W639	OBP15	PBP_GOBP	3	20	15.218
2.79	1.60	A0A088AND4	apidermin 2 precursor		2	35.1	7.7514
2.98	1.31	Q3LBA7	CSP1	OS-D	5	53.4	13.844
4.27	1.25	A0A088AH97	uncharacterized protein	CD36	3	9.8	42.591
2.64	1.08	A0A088AJ72	very long-chain-fatty-acid--CoA ligase bubblegum isoform X1	AMP-binding	5	11.9	76.927
1.84	1.05	Q8I6X7	CSP3	OS-D	3	28.5	14.954
3.03	1.05	A0A088AKG2	Carboxylic ester hydrolase	COesterase	6	13.7	65.688
4.97	1.03	A0A087ZRK9	Catalase	Catalase	12	33.5	55.334
3.00	0.94	A0A087ZSL7	calcyphosin-like protein	EF-hand_7	4	22.4	24.576
2.82	0.93	A0A088AFI9	tubulin alpha-1 chain-like isoform X1	Tubulin	1	30.7	50.205
3.12	0.90	A0A088A285	V-type proton ATPase subunit d	vATP-synt_AC39	2	10.9	39.566
3.69	0.88	A0A088A4D4	sodium/potassium-transporting ATPase subunit beta-2-like	Na_K-ATPase	3	10.5	35.986
2.82	0.73	A0A088AW01	Carboxylic ester hydrolase	COesterase	12	22.6	61.713
2.03	0.71	Q9U9J5	OBP2	PBP_GOBP	4	28.9	15.658
1.83	0.71	A0A088A822	delta-1-pyrroline-5-carboxylate synthase isoform X1	AA_kinase	1	1.5	85.033
6.21	0.68	A0A088A9A9	uncharacterized protein	ERM	16	25	74.599
2.62	0.67	A0A087ZQU9	calnexin isoform X1	Calreticulin	1	3.2	70.225
3.15	0.67	A0A088AUP1	phosphatidylinositol transfer protein alpha isoform	IP_trans	6	31.4	31.549
2.56	0.62	A0A088AFL5	glutathione S-transferase D1 isoform X1	GST_C	4	17.1	27.572
4.71	0.59	A0A087ZVB3	circadian clock-controlled protein-like	JHBP	3	9.8	27.995
2.41	0.59	A0A087ZTA6	Sodium/potassium-transporting ATPase subunit alpha	Cation_ATPase_C	18	21.4	115.3
2.29	0.58	A0A088AS57	uncharacterized protein	EcKinase	11	26.4	48.37
2.91	0.57	A0A088AVX5	NADH dehydrogenase [ubiquinone] 1 alpha subcomplex subunit 2	L51_S25_CI-B8	1	16	10.958
2.40	0.57	A0A087ZQ27	Heat shock protein	HSP70	17	35.9	71.769
2.81	0.53	A0A087ZZ55	Nidogen-1	EGF_3	15	14.2	143.62
3.05	0.51	A0A087ZYD0	Aldehyde oxidase	Ald_Xan_dh_C	26	25.5	140.86
3.43	0.51	A0A088AGI5	Alcohol dehydrogenase [NADP+] A	Aldo_ket_red	13	44.3	36.382
3.58	0.47	A0A088AC27	Alpha-tocopherol transfer protein-like	CRAL_TRIO	15	51.3	32.377
2.33	-0.68	A0A088AMJ8	pro-resilin isoform X2	Chitin_bind_4	3	23	21.422
2.00	-0.75	A0A087ZSC2	uncharacterized protein		1	22.2	10.563
2.33	-1.04	Q86PH6	Transferrin	Transferrin	21	42.3	78.657
2.47	-1.10	A0A088A0P7	uncharacterized protein		1	18.6	10.913
1.64	-1.14	A0A088AQB0	Leucine-rich repeat-containing protein	LRR_1	5	10.6	75.705
1.98	-1.16	A0A087ZXF8	uncharacterized protein		1	3.7	36.466
1.96	-1.17	A0A088AMK2	Chitinase-like protein Idgf4	Glyco_hydro_18	17	53	50.13
2.50	-1.18	A0A088AS56	Apolipophorin	DUF1081	94	33.8	367.61
2.39	-1.32	A0A088AQ33	uncharacterized protein		9	31.7	51.132
3.01	-1.33	A0A088A045	NPC2-1	E1_DerP2_DerF2	2	9.7	16.793
1.87	-1.36	A0A088AG46	uncharacterized protein		2	4.3	60.357
2.02	-1.40	A0A088AQ81	Carboxylic ester hydrolase	COesterase	3	6.6	65.249
2.39	-1.49	A0A088AKR9	Flexible cuticle protein	Chitin_bind_4	4	51.5	14.978
1.45	-1.61	B0LUE8	Apolipophorin-III-like protein	ApoLp-III	8	68.9	21.348
3.78	-1.96	Q1W636	OBP18	PBP_GOBP	1	14.1	15
4.37	-2.04	A0A088AUQ2	uncharacterized protein		4	32.6	9.676
3.30	-2.51	Q1W647	OBP3	PBP_GOBP	2	19.7	17.663
2.49	-2.68	Q868N5	Vitellogenin	DUF1943	2	43.8	201.05
3.59	-2.80	G5D3E9	Vitellogenin	Vitellogenin_N	1	75	24.872
5.62	-3.83	A0A088ADL8	Vitellogenin	DUF1943	81	45.9	200.95
4.50	-4.03	A6YLP7	Hexamerin	Hemocyanin_C	21	31	81.486
4.57	-6.63	A0A088A882	Leucine-rich repeat-containing protein	LRR_8	10	24.4	42.779
