# Supplementary material for: Antennal Protein Profile in Honeybees: Caste and Task Matter More Than Age
Source: Front Physiol. 2018 Jun 20;9:748. doi: 10.3389/fphys.2018.00748 (PMC6019485; doi:10.3389/fphys.2018.00748)
Supplement: TABLE S4 — Soluble olfactory proteins differentially expressed in single groups of the two castes (Student t-test Benjamini Hochberg-corrected FDR = 5%). [file Table_4.PDF]

Uniprot Accession number	Description	Pfam	Comparison	-Log Student's T-test p-value	Student's T-test Test statistic
Q1W647	OBP3	PBP_GOBP	nurses-virgin queens; nurse-mated queens; mated-established queens	2.17; 2.92; 2.57	-5.14; -8.2; 6.62
Q9U9J5	OBP2	PBP_GOBP	nurses-virgin queens	2.02	4.67
A0A088A045	NPC2-1	E1_DerP2_DerF2	nurses-virgin queens; nurse-mated queens	2.18; 2.24	-5.17; -5.37
A0A088A4K9	OBP14	PBP_GOBP	foragers-established queens	3.93	-14.96
Q1W636	OBP18	PBP_GOBP	foragers-established queens	3.02	-8.74
A0A088A1I8	delta-1-pyrroline-5-carboxylate dehydrogenase, mitochondrial	Aldedh	nurses-young queen	2.22	3.88
A0A088AQ81	esterase E4-like	COesterase	nurses-young queen	2.51	-4.42
A0A088AW01	esterase FE4-like	COesterase	nurses-young queen	2.16	3.77

Supplementary Table S4. Soluble olfactory proteins differentially expressed in single groups of the two castes (Student t-test Benjamini Hochberg-corrected FDR=5%).
