# Supplementary material for: Antennal Protein Profile in Honeybees: Caste and Task Matter More Than Age
Source: Front Physiol. 2018 Jun 20;9:748. doi: 10.3389/fphys.2018.00748 (PMC6019485; doi:10.3389/fphys.2018.00748)
Supplement: TABLE S5 — Soluble olfactory proteins differentially expressed in groups of workers (Student t-test Benjamini Hochberg-corrected FDR = 5%). [file Table_5.PDF]

Uniprot Accession number	Description	Pfam	Comparison	-Log Student's T-test p-value	Student's T-test Test statistic
A0A088A4K9	OBP14	PBP_GOBP	nurses-old workers	2.72	4.84
A0A088ABV3	Glutathione S-transferase	GST_C	guards-2nd week	4.10	16.52
A0A088AVD8	peroxisomal multifunctional enzyme type 2-like	adh_short	guards-2nd week	2.66	6.99

Supplementary Table S5. Soluble olfactory proteins differentially expressed in groups of workers (Student t-test Benjamini Hochberg-corrected FDR=5%).
